# Preoperative Blood Glucose Level Predicts Postsurgical Gastroparesis Syndrome after Subtotal Gastrectomy: Development of an Individualized Usable Nomogram

**DOI:** 10.1155/2020/7058145

**Published:** 2020-05-14

**Authors:** Chenchen Mao, Xin Liu, Yunshi Huang, Mingming Shi, Weiyang Meng, Libin Xu, Weisheng Chen, Yuanbo Hu, Xinxin Yang, Xiaodong Chen, Xian Shen

**Affiliations:** ^1^Department of Gastrointestinal Surgery, The Second Affiliated Hospital, Wenzhou Medical University, Wenzhou, Zhejiang, China; ^2^Department of Gastrointestinal Surgery, The First Affiliated Hospital, Wenzhou Medical University, Wenzhou, Zhejiang, China; ^3^Department of Emergency Medical, The Second Affiliated Hospital, Wenzhou Medical University, Wenzhou, Zhejiang, China

## Abstract

**Background:**

Postsurgical gastroparesis syndrome (PGS) after subtotal gastrectomy imposes significant social and economic burdens. We aimed to investigate the relationship between preoperative blood glucose level and PGS and develop a nomogram for individualized prediction. *Patients and Methods*. We retrospectively analyzed 633 patients with gastric cancer who underwent subtotal gastrectomy. Preoperative blood glucose levels were evaluated via receiver operating characteristic (ROC) curve analysis. Chi-squared tests and multivariable logistic regression analyses were used to develop a predictive model for PGS, presented as a nomogram, which was assessed for its clinical usefulness.

**Results:**

Thirty-eight of 633 patients were diagnosed with PGS. Based on the ROC curve analysis, the preoperative blood glucose cutoff value for PGS was 6.25 mmol/L. The predictors of PGS included preoperative hyperglycemia (odds ratio (OR) 2.3, *P* = 0.03), body mass index (BMI; OR 0.21, *P* = 0.14 for BMI < 18.5 and OR 3.0, *P* = 0.004 for BMI > 24), and the anastomotic method (OR 7.3, *P* = 0.001 for Billroth II and OR 5.9, *P* = 0.15 for Roux-en-Y). The predictive model showed good discrimination ability, with a C-index of 0.710, and was clinically useful.

**Conclusions:**

Preoperative hyperglycemia effectively predicts PGS. We present a nomogram incorporating the preoperative blood glucose level, BMI, anastomotic method, and tumor size, for individualized prediction of PGS.

## 1. Introduction

Postsurgical gastroparesis syndrome (PGS) presents with symptoms suggesting gastric retention, including delayed gastric emptying, in the absence of mechanical obstruction [1]. Its prevalence following gastrectomy has been reported to be 0.4% to 5.0% [2]. PGS imposes significant social and economic burdens, including increased hospitalization time and healthcare costs [3]. However, preventive and therapeutic measures, such as dietary restrictions, medications that accelerate emptying, nonpharmacological interventions, and psychological therapies, have been proved to be effective [4, 5]. Therefore, accurate diagnostic methods are required to identify patients at high risk of developing PGS in order to offer targeted prophylactic measures.

Diabetes, which manifests as a group of metabolic diseases characterized by hyperglycemia, has been found to be associated with gastroparesis. A recent questionnaire study reported that 9.8% of individuals with type 1 diabetes experienced symptoms of gastroparesis [6]. The incidence of gastroparesis is reportedly higher in individuals with either type 1 or type 2 diabetes compared with those without diabetes [7]. Furthermore, it has been reported that acute changes in glycemia can impact the rate of gastric emptying. For example, acute hyperglycemia significantly slows gastric emptying in both healthy individuals and those with type 1 diabetes [8, 9], while insulin-induced hypoglycemia markedly accelerates gastric emptying [10]. However, these phenomena have not been well studied, and whether the preoperative blood glucose level is related to PGS remains controversial, with no consensus on the optimal cutoff value.

To the best of our knowledge, no study to date has determined the optimal cutoff value of the preoperative blood glucose level to reduce the rate of PGS. Therefore, our study is aimed at investigating the relationship between preoperative fasting blood glucose level and PGS. Specifically, we sought to develop an individualized usable nomogram for prediction of PGS in patients with gastric cancer.

## 2. Patients and Methods

### 2.1. Patients

A total of 633 patients with gastric cancer who underwent subtotal gastrectomy in the Department of Gastrointestinal Surgery, First Affiliated Hospital of Wenzhou Medical University, China, between January 2009 and December 2012 were enrolled. The study was approved by the Ethics Committees of the First Affiliated Hospital of Wenzhou Medical University, and all participants provided written informed consent prior to participation in the study.

### 2.2. Diagnosis of PGS

PGS was diagnosed according to the following criteria, which were reported in our previous research [2]: ≥1 medical examinations confirming the absence of mechanical gastric outflow obstruction; stomach drainage volume > 800 mL/day sustained for >10 days; no obvious abnormality in fluid-electrolyte balance; no underlying disease, such as hypothyroidism or choroiditis, which may cause PGS; and no current treatment with any medications that may affect smooth muscle contraction.

### 2.3. Blood Glucose Level

Blood glucose levels were obtained when patients were most likely fasting (before 7:30 AM, when breakfast was served in the hospital). Additionally, pre- and postoperative blood glucose levels were obtained the day before and after the operation, respectively. We determined the cutoff value of the blood glucose level as the maximum Youden index value determined by receiver operating characteristic (ROC) curve analysis.

### 2.4. Data Analysis

The Kolmogorov-Smirnov test was performed to assess the distribution equality of the continuous parameters. Normally distributed data are presented as the mean ± SD, whereas nonnormally distributed data are presented as the median and IQR. The independent *t*-test and Mann–Whitney *U* test were used to analyze intergroup differences in continuous variables, while the chi-squared test and Fisher exact test were used for categorical variables. Multivariable logistic regression analysis employing forward stepwise selection was performed using the clinical predictors that were statistically significant in univariate analysis. A nomogram that could quantitatively predict the incidence of PGS was constructed based on the results of multivariable logistic analysis. Decision curve analysis was further conducted to determine the clinical usefulness of the nomogram by quantifying the net benefits at different threshold probabilities in the validation data set. All *P* values were 2-sided, and *P* < 0.05 was considered statistically significant. All statistical analyses were performed using SPSS version 22.0 (SPSS Inc., Chicago, IL, USA) and R version 3.0.1 (http://www.Rproject.org).

## 3. Results

### 3.1. Patient Characteristics

As shown in Table 1, most patients were male (*n* = 454; 71.7%), and 298 of the 633 enrolled patients were aged >65 years (47.7%). A large number of patients (*n* = 383; 62.0%) had a standard weight, with a body mass index (BMI) of 18.5 to 24 kg/m^2^. Based on a preoperative blood glucose cutoff value of 6.25 mmol/L, a large proportion of patients (*n* = 403; 63.7%) were determined to have preoperative hypoglycemia. A majority of patients had tumors smaller than 4.75 cm (*n* = 471; 74.8%), and nearly half had stage III gastric cancer (*n* = 278; 47.7%). Of the 633 patients analyzed, 38 (6.0%) were diagnosed with PGS.

### 3.2. Blood Glucose Characteristics

**Figure 1:**
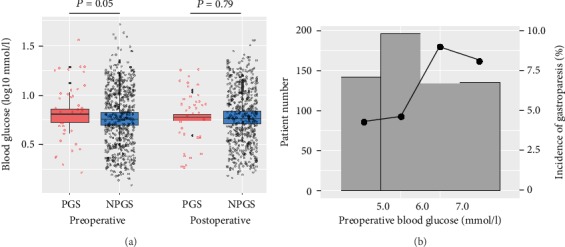
Blood glucose and PGS characteristics. (a) Distribution of preoperative and postoperative blood glucose between PGS and NPGS. (b) Frequency distribution of patients and PGS incidence of different blood glucose strata.

As shown in Figure 1(a), compared with patients without PGS, those with PGS had significantly higher preoperative blood glucose levels (*P* = 0.05). However, these groups did not differ significantly in terms of the postoperative blood glucose level (*P* = 0.79). Further, there was an overall linear increase in the rate of PGS as the preoperative blood glucose level increased (Figure 1(b)).

### 3.3. Variables Associated with PGS

Based on ROC curve analysis, the cutoff values of pre- and postoperative blood glucose levels for PGS were 6.25 and 5.65 mmol/L, respectively. Patients were further grouped according to these cutoff values.

The chi-squared test was used to examine the relationships between clinical characteristics and PGS. In univariate analysis (as shown in Table 1), BMI (*P* = 0.001), hypertension (*P* = 0.01), preoperative blood glucose level (*P* = 0.001), Charlson Comorbidity Index (*P* = 0.01), tumor size (*P* = 0.03), and anastomotic method (*P* = 0.001) are significantly correlated with PGS (Table 1). These factors were subsequently included in multivariate logistic regression analysis, which identified the preoperative blood glucose level (odds ratio (OR) 2.3 (95% CI, 1.1-4.8), *P* = 0.03), BMI (<18.5 kg/m^2^: OR 0.21 (95% CI, 0.03-1.6), *P* = 0.14; >24 kg/m^2^: OR 3.0 (95% CI, 1.4-6.3), *P* = 0.004), and anastomotic method (Billroth II: OR 7.3 (95% CI, 2.2-24.8), *P* = 0.001; Roux-en-Y: OR 5.9 (95% CI, 0.54-65.5); *P* = 0.15) as independent predictors (Table 2).

### 3.4. Nomogram Development and Usefulness

**Figure 2:**
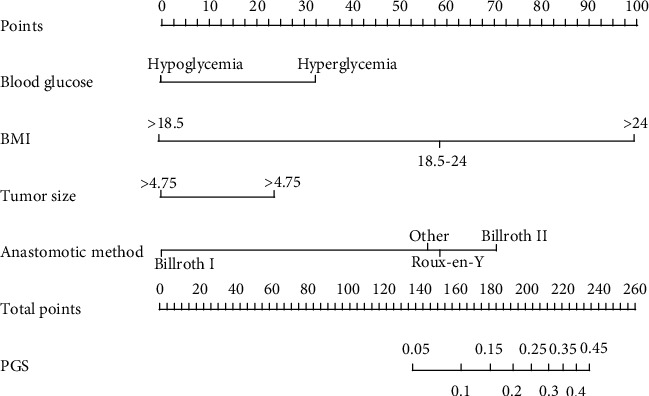
Developed nomogram.

**Figure 3:**
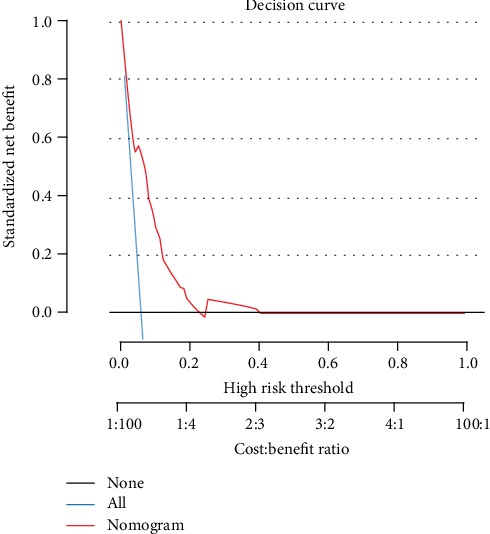
Decision curve analysis for the nomogram. The *y*-axis measures the net benefit. The red line represents the nomogram. The blue line represents the treat-all scheme, and the black line represents the treat-none.

A model incorporating the independent predictors (*P* < 0.1 in multivariate logistic regression analysis) was developed and presented as a nomogram (Figure 2). The nomogram showed good discrimination with a C-index of 0.710. Decision curve analysis for the nomogram is presented in Figure 3.

Decision curve analysis demonstrated that if the threshold probability of a patient is 1%, using the nomogram adds more benefit than either the treat-all or treat-none scheme. However, when the probability of a patient is 23% to 24%, no increased benefit was found. For example, if the personal threshold probability of a patient is 10% (i.e., the patient would opt for treatment if his/her probability of PGS was 10%), then the net benefit when using the nomogram to make the decision of whether to undergo treatment is 0.287, showing added benefit compared with the treat-all or treat-none scheme.

## 4. Discussion

PGS is a disorder of the gastrointestinal tract and a common complication after abdominal surgery. Although PGS is commonly scintigraphically measured by employing 99mTc-sulfur colloid bound to solid food, newer technologies also show promise [1]. A method using a meal of radiolabeled toast, jam, and low-fat egg substitute was endorsed as a diagnosis for gastroparesis, with gastric retention > 60% at 2 hours and/or >10% at 4 hours [11]. Another study [12] reported that a wireless motility capsule that quantifies changes in intraluminal pH and pressure offers an alternative to scintigraphy. Moreover, nonradioactive ^13^C-urea breath tests that quantify exhaled ^13^CO_2_ after duodenal assimilation of a standardized substrate can also be used as an alternative to scintigraphy [13]. However, such methods may be unreliable with abnormal digestive function, and further improvements in diagnostic testing and prediction of PGS are anticipated.

Diabetes has been reported to be associated with gastroparesis in previous studies [1, 14]. However, our study showed that although diabetic patients with gastric cancer were more likely to develop PGS compared with their nondiabetic counterparts, the difference was not significant. Considering that most diabetic patients may control their blood glucose with hypoglycemic drugs or insulin, we hypothesize that it is the blood glucose level rather than the diabetes that is involved in the development of PGS. In view of the Warburg effect, which involves high uptake of glucose, enhanced glycolysis, and changes in glycometabolism, exhibited in gastric cancer epithelial cells [15] and influenced by surgical stressors, both pre- and postoperative blood glucose levels are greatly changed. Therefore, we used the blood glucose cutoff values of preoperative PGS-related hyperglycemia (≥6.25 mmol/L) and postoperative PGS-related hyperglycemia (≥5.65 mmol/L), as determined by ROC curve analysis, rather than the general threshold of 6.1 mmol/L. As a result, 230 (36.3%) and 321 (56.1%) patients were considered to have pre- and postoperative hyperglycemia, respectively. Interestingly, further research demonstrated that preoperative hyperglycemia was an independent risk factor for PGS, whereas postoperative hyperglycemia was not.

The pathophysiologic mechanism by which hyperglycemia leads to PGS is likely to be multifactorial. Unequivocal evidence has demonstrated that hyperglycemia is associated with reduction of antral contractions and stimulation of pyloric contractions, as well as dysregulation of antroduodenal function [8, 16], which leads to reversible slowing of gastric emptying. These changes in gastric emptying are likely to represent an additional level of glucose regulation: entry of glucose into the small intestine is slowed at times of relative hyperglycemia and accelerated to mitigate hypoglycemia [17]. Emerging evidence supports that the effect of hyperglycemia slowing gastric emptying might also be related to putative mediators, such as ghrelin and nitric oxide [18, 19]. On the other hand, surgical stress and reconstruction of the digestive tract impact blood glucose directly and change gastric electrical activity greatly, resulting in loss of diagnostic specificity of postoperative hyperglycemia for PGS.

Similar to our previous study that demonstrated visceral obesity as an independent risk factor for PGS [2], we found that overweight/obese patients (defined by BMI > 24 kg/m^2^) were more likely to develop PGS and that overweight/obesity was an independent risk factor. It is well known that obesity is usually accompanied by changes in glucose metabolism. Thus, the effect of obesity promoting PGS might be secondary to an abnormal blood glucose level. Same as our previous study and other research [2, 20], we also found that patients undergoing Billroth II reconstruction were more likely to develop PGS compared with other types of reconstruction. Regardless of the surgical technique, Billroth II reconstruction alters the digestive tract such that it can no longer efficiently break down contractive chime and reduces gastrointestinal smooth muscle contractility.

Considering that only 3 independent risk factors demonstrated a standard of *P* < 0.05, which may reduce the benefit of the predictive model, we relaxed the standard to *P* < 0.1. Thus, a nomogram incorporating the preoperative blood glucose level, BMI, tumor size, and anastomotic method was developed to predict individual PGS incidence. Patients with high risk of developing PGS may be supervised to preventively adopt dietary measures or take medications that accelerate emptying, as a recent survey noted that gastroparetics ingested 1.4 meals daily and only 13% complied with fat restrictions [21]. With this aim, decision curve analysis, which offers insight into clinical consequences based on threshold probability, from which the net benefit can be derived, was applied in this study. The net benefit was defined as the proportion of true positives minus the proportion of false positives, weighted by the relative harm of false-positive and false-negative results. Decision curve analysis showed that if the threshold probability of a patient is >1% and <40%, as determined by the nomogram in the current study, offering prophylactic measures adds more benefit than either the treat-all or the treat-none scheme.

Our study still has several limitations. The first is the small sample size from a single institution. Thus, a larger, multicenter cohort study is needed. Furthermore, as this is a retrospective study, research with a prospective design is essential to further test the benefits of the nomogram before it is adopted in a routine practice.

## 5. Conclusion

This is the first study to determine the relationship between preoperative blood glucose level and PGS, showing that preoperative hyperglycemia effectively predicts PGS. A nomogram incorporating the preoperative blood glucose level, BMI, anastomotic method, and tumor size was developed and is believed to be economical, reliable, and convenient, with high sensitivity and specificity, thus facilitating early implementation of preventive and therapeutic measures.

## Figures and Tables

**Table 1:** Demographics and patient characteristics in the overall study population and by the PGS group.

Factors	Total (*N* = 633)	Gastroparesis group (*N* = 38)	Nongastroparesis group (*N* = 595)	*P*
Age (y)				0.09
>65	298 (47.7)	23 (60.5)	275 (46.4)	
≤65	333 (52.3)	15 (39.5)	318 (53.6)	
Gender				0.78
Male	454 (71.7)	28 (73.7)	426 (71.6)	
Female	179 (28.3)	10 (26.3)	169 (28.4)	
BMI (kg/cm^2^)				0.001
<18.5	110 (17.8)	2 (5.4)	108 (18.6)	
18.5-24	383 (62.0)	19 (51.4)	364 (62.7)	
>24	125 (20.2)	16 (43.2)	109 (18.7)	
Hypertension				0.01
Yes	133 (21.0)	14 (36.8)	119 (20)	
No	500 (79.0)	24 (63.2)	476 (80)	
Preoperative blood glucose (mmol/L)				0.001
>6.25	230 (36.3)	23 (60.5)	207 (34.8)	
≤6.25	403 (63.7)	15 (39.5)	388 (65.2)	
Postoperative blood glucose (mmol/L)				0.08
>5.65	321 (56.1)	26 (70.3)	298 (55.4)	
≤5.65	251 (43.9)	11 (29.7)	240 (44.6)	
Diabetes mellitus				0.15
Yes	40 (6.3)	5 (13.2)	35 (5.9)	
No	593 (93.7)	33 (86.8)	560 (94.1)	
Charlson score				0.01
0	388 (61.3)	16 (42.1)	372 (62.5)	
≥1	245 (37.1)	22 (57.9)	223 (35.8)	
History of abdominal operation				0.26
Yes	59 (9.3)	6 (15.8)	53 (8.9)	
No	574 (90.7)	32 (84.2)	542 (91.1)	
Preoperative obstruction				0.24
Yes	92 (14.5)	8 (21.1)	84 (14.1)	
No	541 (85.5)	30 (78.9)	511 (85.9)	
Preoperative bleeding				0.53
Yes	125 (19.7)	6 (15.8)	119 (20)	
No	508 (80.3)	32 (84.2)	476 (80.0)	
Preoperative perforation				1.00
Yes	2 (0.3)	0 (0)	2 (0.3)	
No	631 (99.7)	38 (100)	593 (99.7)	
Histological classification				0.59
Ulcer	442 (69.8)	28 (73.7)	414 (69.6)	
Nonulcer	191 (30.2)	10 (26.3)	181 (30.4)	
Differentiation types				0.79
Differentiated	554 (87.5)	34 (89.5)	520 (87.4)	
Nondifferentiated	49 (7.7)	3 (7.8)	46 (7.7)	
Signet ring	30 (4.8)	1 (2.7)	29 (4.9)	
TNM stage				0.05
I	183 (31.4)	12 (31.6)	171 (31.4)	
II	56 (9.6)	12 (31.6)	44 (8.1)	
III	278 (47.7)	10 (26.3)	268 (49.2)	
IV	66 (11.3)	4 (10.5)	62 (11.3)	
Tumor size (cm)				0.03
>4.75	158 (25.1)	15 (40.5)	143 (24.3)	
≤4.75	471 (74.8)	22 (59.5)	446 (75.7)	
Anastomotic method				0.001
Billroth I	239 (37.8)	4 (10.8)	235 (39.5)	
Billroth II	351 (55.5)	32 (86.5)	319 (53.6)	
Roux-en-Y	16 (2.5)	1 (2.7)	15 (2.5)	
Other	26 (4.2)	0 (0)	26 (4.4)	

Data are presented as *n* (%). Abbreviations: BMI: body mass index; TNM: tumor-lymph, node, metastasis.

**Table 2:** Multivariate analysis to evaluate potential predictive factors for gastroparesis.

Factors	Multivariate analysis		
	OR	95% CI	*P*
Preoperative blood glucose (mmol/L)			
≤6.25	1		
>6.25	2.3	1.1-4.8	0.03
BMI (kg/m^2^)			
18.5-24	1		
<18.5	0.21	0.03-1.6	0.14
>24	3.0	1.4-6.3	0.004
Hypertension			
No	1		
Yes	1.7	0.60-4.5	0.33
Charlson score			
0	1		
≥1	1.4	0.52-3.7	0.68
Tumor size (cm)			
>4.75	1		
≤4.75	1.9	0.91-4.1	0.09
Anastomotic method			
Billroth I	1		
Billroth II	7.3	2.2-24.8	0.001
Roux-en-Y	5.9	0.54-65.5	0.15
Other	5.3	0.50-56.1	0.16

Abbreviations: BMI: body mass index.

## Data Availability

The data used to support the findings of this study are included within the article.
